# Retraction: Mechanisms Contributing to the Generation of Mayer Waves

**DOI:** 10.3389/fnins.2021.793064

**Published:** 2021-11-01

**Authors:** Frontiers Editorial Office

The journal and Chief Editors retract the 10 July 2020 article cited above.

Following publication, the Karolinska Institutet contacted the editorial office stating that the authors have never been affiliated with it, and contrary to the statement in the article did not receive funding for this work. The authors did not respond to the journal's enquiries and attempt to verify authorship of this article. Therefore the editors no longer have confidence in the integrity of this work.

This retraction was approved by the Chief Editors of Frontiers in Neuroscience and the Chief Executive Editor of Frontiers.

